# Barriers to, and facilitators of, the adoption of a sugar sweetened beverage tax to prevent non-communicable diseases in Namibia: a policy landscape analysis

**DOI:** 10.1080/16549716.2021.1903213

**Published:** 2021-04-20

**Authors:** Hans Justus Amukugo, Safura Abdool Karim, Anne Marie Thow, Agnes Erzse, Petronell Kruger, Abel Karera, Karen Hofman

**Affiliations:** a Community Health Department, School of Nursing, Faculty of Health Sciences, University of Namibia, Windhoek, Namibia; b University of the Witwatersrand, School of Public Health, Johannesburg, South Africa; c Menzies Centre for Health Policy and Director of Academic Titles, School of Public Health, the University of Sydney, Australia; d Allied Health Department, School of Nursing, Faculty of Health Sciences, University of Namibia, Windhoek, Namibia

**Keywords:** Fiscal policy, nutrition-related NCDs, policy analysis, political economy

## Abstract

**Background**: Nutrition-related non-communicable diseases contribute to approximately half of the premature deaths in Namibia. Westernisation and urbanisation of communities have resulted in changing dietary patterns that see people eating more refined and high sugar content foods that are a risk for nutrition-related non-communicable diseases. Sugar-sweetened beverage taxation has been found to influence consumer purchasing behaviour and to raise revenue for health-promoting activity in other low- and middle-income countries.

**Objectives**: To analyse Namibia’s non-communicable diseases prevention policy landscape and assess the readiness of the Government to adopt sugar-sweetened beverage taxation policies for public health.

**Methods**: Government policy documents relating to nutrition-related non-communicable diseases were analysed, utilising predetermined variables based on policy theory. Thirteen key informant interviews were conducted with stakeholders from Government, non-governmental organisations and academic institutions. Data sets were analysed utilising Kingdon’s analytical theory for agenda setting.

**Results**: Nutrition-related non-communicable diseases are an increasing problem that requires immediate action. Diet and lifestyle are recognised as major contributors to non-communicable diseases. The Government has adopted a multisectoral approach to the control and prevention of non-communicable diseases in Namibia. A sugar-sweetened beverage tax is envisaged in policy, but there is no progress towards its enactment. At the highest level of Government, the Ministry of Finance has ruled out immediate action towards sugar-sweetened beverage taxation. There is little publicly available information about the Namibian beverages industry, but it is closely tied to the South African drinking industry and is influenced by policy action in that country.

**Conclusion**: The Government of Namibia has taken positive steps and the policy environment is friendly towards an SSB tax. The proximity of trade and the competitive nature of the Namibian drinks industry with South Africa suggest that a regional perspective to advocacy would be of value.

## Background

Namibia is a middle-income economy with relative political stability [1]. Its major trade partners are South Africa (total exports: 15%; total imports: 44%), China (total exports: 16%; total imports: 5%), Zambia (total imports: 14%), and Botswana (total exports: 9%; total imports: 4%) [2]. Namibia is expected to see an increase of 6.2% in food sales from 2019 to 2022; 9.6% to 11.5% in the sale of sugar products [3], and 4.5% to 6.3% in soft drink sales [3]. Namibia Breweries Ltd., which produces alcohol and soft drinks, and Consolidated Sugar Industries (Namibia) (Pty) Ltd. (sugar producer) are the biggest market players in the Namibian food and drink industry [3].

As ultra-processed foods are becoming a major growth component in sub-Saharan Africa, including Namibia, urbanisation-led changing dietary patterns result in the consumption of more refined products, including sugar sweetened beverages (SSBs) [4]. The over-consumption of SSBs has been shown to increase the prevalence of overweight and obesity, and related non-communicable diseases (NCDs) [5,6]. In Namibia, NCDs are among the top 10 and top 15 causes of morbidity and mortality, respectively [7]. In 2018, 50% of deaths in Namibia were due to NCDs, including cardiovascular diseases, diabetes mellitus, cancers and chronic obstructive airway disease [8].

Namibia is a signatory to a number of international initiatives that recognise that an unhealthy diet with high sugar consumption is a risk factor for NCDs. These include the Moscow Declaration on NCDs (May 2011) [9], the UN Political Declaration on NCDs (September 2011) [10], and the Global Action Plan for the Prevention and Control of Non-communicable Diseases 2013–2020 [11]. These initiatives recommend policy action to reduce sugar consumption [8–10]. One policy intervention that has been shown to reduce obesity is taxation of SSBs [12,13]. The World Health Organization (WHO) has repeatedly endorsed SSB taxation as an evidence-based mechanism to reduce sugar consumption and, in turn, the prevalence of obesity and diabetes [14,15]. SSB taxation seems particularly effective in changing consumer behaviour in lower income groups, which are often more vulnerable to NCDs [13]. Evidence from Mexico shows that consumers are likely to substitute SSBs with water when an SSB tax is imposed [16]. Furthermore, this tax may indirectly reduce state healthcare expenditure, especially in developing countries where resources are limited [17]. Revenue generated through SSB taxation can be directed towards the promotion of healthcare programmes to raise awareness about NCDs, and subsidies for healthier alternatives [15]. In South Africa, the SSB tax has already had an impact on consumer behaviour and the beverage industry. There has been a reported decrease in the consumption of sweetened beverages with an associated gradual uptake of reformulated products since 2018 when South Africa adopted it SSB tax [18]. Industry players have had to reformulate and redesign their products to reduce the sugar content to avoid taxation [18]. However, the political economy of adopting an SSB tax is complex and can face significant challenges [19].

Currently, there is no SSB taxation in Namibia nor are there any immediate plans for its introduction [20], although neighbouring countries are either considering or have implemented an SSB tax.

The objective of this study was to identify and analyse barriers to, and facilitators of, the adoption of SSB taxation policies in Namibia. We undertook a policy analysis with a focus on policies relevant to nutrition-related non-communicable diseases (NR-NCDs) and SSB taxation, as well as stakeholder and contextual factors that may influence the adoption of such policies. This research was part of a larger study undertaken in a subset of eastern and southern African countries: Rwanda, Botswana, Kenya, Namibia, Uganda and Zambia.

## Methods

We conducted a prospective qualitative policy analysis, comprising a desktop review and key informant interviews. The theoretical basis and methodological approach for the documentary data collection and analysis is described in the study design paper [21]. Overall, the Walt and Gilson policy health policy analysis triangle was used to guide data collection [22]. Nine contemporary Government policy documents were reviewed, ranging from policies on health to those on taxation. The selection of policies was determined by the desk-based review and through interviews with persons directly or indirectly involved in policy development and implementation in Namibia. Policies dated 2013–2018, but some policies will continue to apply in future with the period covered extending to 2028. Relevant information from policy documents, including content relevant to NR-NCD prevention, framing, gender and implementation, was extracted.

We utilised Varvasovsky and Brugha’s approach to stakeholder analysis [23] to identify stakeholders from the SSB industry, public health, finance (taxation), and education and health policy (prevention, monitoring and control of NCDs), using web searches and citations in relevant documents. We then mapped corporate political activity of industries relevant to SSBs, using the framework developed by Mialon et al. [24].

We also conducted semi-structured interviews with 13 purposively selected key informants in February 2019, to explore the policy landscape. Respondents were recruited based on their role in nutrition and SSB-relevant policies or the policy landscape. They included participants from national Government in the public health, economic/industry, and social welfare sectors (*n* = 9); economic/industrial sector NGO (*n* = 1), and academic public health departments of tertiary education institutions (*n* = 3) (Table 1). Industry players were invited to participate but did not respond.

**Table 1.** Key informants by sectoral interest and type of organisation (*N* = 13)

Type of organisation	Sectoral interest of respondents^a^
National Government (*n* = 9)	Public health (*n* = 6) Economic/trade and industry (*n* = 2) Social welfare (*n* = 4)
NGO (*n* = 1)	Economic/industry (*n* = 1)
Academic institution (*n* = 3)	Public health (*n* = 3)

^a^Three national Government respondents had dual responsibilities.

Interview questions were drawn from Kingdon’s theory of agenda-setting [25] and included questions about (i) perceptions of NR-NCDs and nutrition as problems in Namibia; (ii) stakeholder opinions regarding policy ‘solutions’ to NR-NCDs, including leveraging an SSB tax; (iii) the potential uses of future taxation revenue with respect to increased access to healthy foods, and; (iv) the potential enablers of, and barriers to, developing and strengthening NR-NCD policy across the sectors. The interviews were audio-recorded and transcribed verbatim; each lasted 30–60 minutes.

## Ethical considerations

This study was approved by the Ministry of Health and Social Service (MoHSS), reference number 17/3/3 HJA; Ethical clearance was obtained from the University of Namibia, Research Ethics Committee (clearance number SOPH/434/2018). Written consent to record interviews was obtained from respondents after the study was fully explained. Permission was also secured from the institutions from where respondents were recruited. All respondents participated on the basis of anonymity.

### Data analysis

The qualitative interview data were coded using predetermined themes based on the study frameworks [18]. The focus of the analysis was on what policy change might entail (current policy context), who might be critical in policy change (actors and institutions), and how policy change might be influenced. The main themes were: influential institutions and actors, existing policies, policy context, factors influencing policy development, factors influencing policy implementation, strengths in existing policies, opportunities/gaps in existing policies, and barriers to policy change, facilitators to policy change, and actor roles and interests. The coded interview data and the documentary data in the matrixes were analysed in combination, using Kingdon’s analytical framework [22]. This integrated analysis focused on the political context, the nature of the (perceived) policy problem, and SSB taxation as a potential policy ‘solution’ [22].

## Results

The findings are presented, using the three categories in Kingdon’s theory of agenda setting: the problem, existing policy, and stakeholder politics [22]. In each category, the facilitators of, and barriers to, changes were identified as they related to the adoption of SSB taxation. Table 2 provides a summary of the major findings.

**Table 2.** Summary of findings using three categories of Kingdon’s theory of agenda setting

Kingdon category	Facilitator of SSB taxation	Barrier to SSB taxation
*Category 1: Understanding the problem of NR-NCDs and SSB taxation*	Government has acknowledged the scope and impact of the problem of NR-NCDs through various policies. A multisectoral approach contributes to effective solution-making.	Unhealthy diets persist with foods with high sugar content, and excessive calorie consumption. Low literacy, poverty and unemployment result in healthy food choices being secondary to other needs. The absence of detailed and granular information about NCDs.
*Category 2: Policy related to NR-NCDs and SSB taxation*	Policies emanate from several sectors. SSB tax is envisaged in policy.	Several different approaches to the problem have been postulated. Interviewees believe solutions should be ‘gender-neutral’. Resources to combat SSBs are not clear in existing policy documents.
*Category 3: Politics of major stakeholders*	High-level political acknowledgement of NR-NCDs. Commitment to international and regional treaties. Regional trade partners have adopted an SSB tax	Influence of industry stakeholders is unknown. Perception that NCD management would be in conflict with other socio-economic development goals.

### Category 1: understanding the problem of NR-NCDs and SSB taxation

The problem of NR-NCDs is well recognised in Namibia, although the contribution of SSBs to the problem of NR-NCDs is less understood. The Government of Namibia’s Vision 2030 and Health Policy Framework both identify the need for action regarding NCDs, including increasing awareness and health promotion at all levels [26,27].

The most important policy statement is the National Multisectoral Strategic Plan for Prevention and Control of Non-Communicable Diseases (NCDs) in Namibia 2017/18 – 2021/22 [28]. This strategic plan recognises cardiovascular disease, stroke, diabetes mellitus and cancer as major NCDs. It emphasises the burden that NCDs place on the healthcare system through increased morbidity, mortality and disability, and that they require urgent action, as well as their negative impact on social development, such as exacerbating inequalities between populations [25]. Related policy and key informant interviews confirmed that the Government of Namibia and other stakeholders recognised NCDs as an emerging problem that required immediate action in several sectors.

The Plan discusses the use of tobacco and tobacco products, harmful use of alcohol, lack of physical exercise, and unhealthy diets as the main behavioural risk factors contributing to the rise in NCDs in Namibia. Although the Plan identified unhealthy diet as a contributor to NCDs, the contribution of SSBs was not explicit. The Plan envisages using food quality standards and better food labelling, and limits on levels of fat, sugar and salt as avenues to improve consumer habits [25]. Although the Plan does not recognise or describe SSBs as a major problem, it does recommend an SSB tax to reduce sugar intake. However, no progress has been made towards the formulation or introduction of such tax. Interviewees indicated that SSBs were a major contributor to NCDs, given the high sugar content and excessive consumption of SSBs in Namibia.

The issue of limited data availability was reported as a major problem by respondents. They expressed concern that the lack of reliable data on the prevalence, mortality and diet-related risk factors for NCDs in Namibia hinders policy planning, and monitoring and evaluation efforts. This concern was supported by the findings from the desk-based research. For example, prevalences of diabetes and hypertension, and correlations with gender, were poorly described in the Demographic Health Survey (DHS) of 2013. This is striking as the DHS included several other detailed population demographic breakdowns, e.g. age of the population, age at marriage, and household age. Malnutrition in Namibia was described by difference in age for stunting and malnutrition [29]. However, data on the prevalence of different NCDs, household food expenditures, and the differences between rural and urban populations in Namibia were not available. Respondents recommended further research to better inform policy and action towards prevention and control of NCDs.

Disparities in access to healthy food and lifestyles due to socioeconomic factors, such as levels of education and literacy, poverty and unemployment, were raised by respondents as common underlying reasons that limit individual options and choice when selecting what food to consume. These factors make decisions regarding healthy food choices for many Namibians secondary to other competing priorities, such as securing accommodation.

The multisectoral approach adopted by the Government of Namibia was often poorly coordinated, had limited financial resources, and did not have sufficient stand-alone technical capacity [30]. Respondents also indicated the need to involve several sectors to adequately address NCDs.

### Category 2: policy related to NR-NCDs and SSB taxation

Table 3 summarises the major policy documents reviewed. These included Government-wide strategies, such as the National Development Plan and sectoral-specific policies in health, agriculture, finance and trade, and industry.

**Table 3.** Namibian policy documents relating to NR-NCDs and nutrition

Document	Content related to NCDs and nutrition	Responsibility ministry
*Namibia’s 5th National Development Plan (NDP5) 2017–2022*	Increase productivity in agriculture and improve nutrition; Government will procure locally produced food and urges other organisations to do so.	Ministry of Economic Planning Ministry of Agriculture, Water and Forestry
*National Multisectoral Strategic Plan for Prevention and Control of Non-Communicable Diseases (NCDs) in Namibia 2017/18 – 2021/22*	Government will provide the fiscal environment and policies necessary for NCD prevention and control; reduce sugar consumption through effective taxation of SSBs.	Ministry of Health and Social Service Ministry of Finance Ministry of Industrialisation, Trade and Small & Medium Enterprise (SME) Development
*Ministry of Health and Social Service – Strategic Plan (2017/2018 – 2021/2022)*	Refer to implementation of programmes that aim at reducing NCDs; refer to role of Ministry of Agriculture to ensure sufficient food and nutritional security.	Ministry of Health and Social Services Ministry of Agriculture, Water and Forestry Ministry of Finance
*Nutrition guidelines for prevention and management of non-communicable diet related diseases January 2013*	Recommends limiting daily intake of sugar.	Ministry of Health and Social Services
*National Food and Nutrition Security Policy 2018–2028*	One of the policy objectives strengthens local production of safe and nutritious foods, especially by smallholder farmers.	Office of Prime Minister Ministry of Agriculture, Water and Forestry Ministry of Health and Social Services Ministry of Information and Communication Technology
*Agriculture Marketing, Trade Policy and Strategy 2011*	Namibia aims to ensure that all agricultural and agro-industrial products both imports and Namibian originating products – destined for the domestic market meet the set minimum quality standards, technical regulations and food safety requirements.	Ministry of Agriculture, Water and Forestry Ministry of Health Social Services Ministry of Information and Technology Ministry of Finance Ministry of Trade and Industry
*Namibia Food Safety Policy 2015*	The Namibia Standards Institution under the Ministry of Industrialization, Trade and SME Development is responsible for implementing the Standards Act (2005), which controls standards such as additives, processing aids, and all products traded in Namibia.	Ministry of Agriculture, Water and Forestry Ministry of Health and Social Services Ministry of Fisheries and Marine Resources Ministry of Industrialization, Trade and SME Development
*Value-Added Tax Amendment Act 12 of 2015*	Prescribes different taxation rates for food and drink products imported into Namibia.	Ministry of Finance Ministry of Trade and Industry
*Namibia Vision 2030: a vision for Namibia*	Create access to abundant, hygienic and healthy foods, based on food security.	All Government ministries and agencies
*National health policy framework 2010–2020*	Sets general priorities for public health, which include nutrition and NCDs	Ministry of Health Social Services and all stakeholders in health and social services delivery

The prevention and control of NCDs is a priority for the Namibian Government; co-ordination is through the Prime Minister’s office. Table 3 shows that NCD and nutrition-related policy is found in health, agriculture, finance and trade sectors, but that the health sector is mainly responsible for NCD prevention and control. The key priority of the health sector is to reduce the preventable and avoidable burdens of morbidity, mortality and disability due to NCDs in the country, and to achieve a healthy and productive population [25].

The different policies in Namibia present varied approaches to NCD prevention and control. In the health sector, these include individual-driven solutions such as providing nutritional and exercise recommendations based on specific NCDs, and drawing inspiration from successful mechanisms in tobacco control legislation [27,31]. Broader policy interventions include promoting minimum food safety and domestic agriculture, which could serve as a check on food quality and incentives for fresher food products [32].

The Strategic Plan outlines the following important principles or approaches to NCDs, which are inclusive and wide ranging: specifically, prevention and control of NCDs should be equity-based and responsive to race, gender, language, religion, opinion and socioeconomic status; Government should respond via different ministries, but individuals, families, NGOs and bilateral development partners should also contribute to empowering healthy environments for communities; and efforts should both be preventive and responsive, and based on scientific evidence and best practice [20].

The Plan’s key action items include tax and subsidies to both discourage certain habits, such as taxation on SSBs to reduce sugar consumption, and encourage healthy food choices; regulations on salt, certain fats and sugar, and food labelling and marketing, especially for children; and mass media campaigns about healthy diets and ensured availability and affordability of healthy foods in schools, educational institutes and the workplace [20]. The action items are formulated in basic terms and require more detailed policy explanations and implementation. Currently, SSBs have no specific tax provision and are only levied under the Namibia VAT Act.

The Namibian National Development Plan [33] clearly directs policy originators to ‘Mainstream gender in all sector policies, programmes and budgets’, but evidence from the policy documents show that this happens to varying degrees in practice. The National Multisectoral Strategic Plan for Prevention and Control of Non-Communicable Diseases in Namibia recognised the issue of gender in prevention and control of NCDs. The Plan clearly specifies that the Government will give special attention to gender equity during development of NCD prevention and control plans and processes. Interviewed respondents were also of the opinion that policies and action towards prevention and control of NCDs should be gender-inclusive as their perceptions were that NCDs affect everyone. Only two respondents were of the opinion that women might be excluded from NCD strategies if gender sensitivity was excluded in decision-making and policy action.

### Category 3: politics of major stakeholders

Our stakeholder analysis (Table 4) findings were that some stakeholders were in favour of, and others opposed, SSB taxation, and many stakeholders were perceived as having a high level of influence over NCD prevention policy. Overall, ministerial mandates seem to determine the SSB taxation position of different Government ministries.

**Table 4.** SSB-related taxation and level of influence – stakeholder analysis findings

Stakeholder	Reason for interest in SSB-related taxation	Perceived level of influence
Office of the Prime Minister (OPM)	To reduce the preventable and avoidable burden of morbidity, mortality and disability due to NCDs in the country, and achieve a healthy and productive population	High, due to coordinator function
Ministry of Health and Social Services	1. To reduce the preventable and avoidable burden of morbidity, mortality and disability due to NCDs in the country, and achieve a healthy and productive population 2. To reduce modifiable risk factors for NCDs and underlying social determinants through the creation of health promoting environments	High, due to interest in health
Ministry of Industrialization, Trade and SME Development	To reduce burden of NCDs on the population and workforce To ensure economic growth and investor confidence	High, due to potential effect on industries
Ministry of Agriculture, Water and Forestry	To ensure increased interest and access to healthy food To increase agriculture production of healthy foods	Medium
Ministry of Finance	To generate revenue through taxation and punitive legislation	High, due to possibility of revenue generation
Ministry of Information, Communication and Technology	To ensure that correct and appropriate health information is disseminated to the population with regard to SSB and NCDs	Low, as they have no direct benefit
Academic institutions	To generate evidence through research to support improvement in public health and reduce burden of NCDs	Medium
Industry	To ensure SSB products remain on the market and generate profit	High, to remain viable

The Government of Namibia initiated the Multi-sectorial Strategic Plan for Prevention and Control of Non-Communicable Diseases in Namibia, which links all concerned stakeholders and aims at consolidating individual effort to maximize gains at reducing NCDs. This was in line with the recommendation of the 2011 Political Declaration on NCDs targeting the multifaceted origins of NCDs [34]. The coordination of this response to NCDs is housed within the executive arm of Government in the Prime Minister’s office, showing further commitment by the Namibian government to prioritising control of this problem. This can be regarded as a positive move by the Government as it plays a central role and can influence all actors concerned with the problem. The Namibian Government demonstrated its openness to outside influence. For example, the Strategic Plan endorses the WHO’s ‘best buys’, which are high-impact interventions aimed at combatting NCDs [35]. The Government has committed to several international instruments aimed at combatting NCDs [36].

Regionally, Namibia’s proximity and political ties to South Africa has led to a significant trade relationship. South Africa is Namibia’s largest trade partner; previously, Namibia’s sugar supply was provided almost exclusively by South Africa. However, due to an oversupply of sugar from world markets and the strengthening Namibian dollar, Namib Mills decreased its sugar prices by 11% to 16% in 2018. This triggered a similar reduction in prices by sugar producers in South Africa [37]. In 2018, Namibia imported approximately N$439 million worth of non-alcoholic beverages from South Africa [26]. The two countries also influence each in the beer market, spurred by competing and highly profitable beer companies [3]. Both countries form part of the Southern Africa Customs Union which provides for the largely free movement of goods between member states, in terms of both physical transit, and zero rating of tax rates for customs, with certain products exempted, such as sugar [38]. The Union also attempts to harmonize marketing techniques to impact production and consumption of agricultural goods, such as sugar. It is therefore interesting that, despite the recent introduction of an SSB tax in South Africa, and the policy undertaking to introduce such a tax locally in the multisectoral Strategic Plan by the Namibian Prime Minister, the Minister of Finance, in response to announcement of the South African SSB tax in 2016, publicly commented that the Namibian Government would not be introducing a SSB tax due to economic challenges [39]. He did not categorically denounce such a tax, but indicated that a more phased approach, with interim measures, such as reducing the zero-rating of sugar, might be more immediately feasible [11]. This demonstrated that Government stakeholders are not united on SSB taxation. It speaks to either a disjoint between policies or a non-committal attitude to action at the highest levels. Some respondents believed that, on an entirely practical plane, due to the high level of imports, South African price increases will have a ripple effect in Namibia [10].

The SSB and related industries are another important stakeholder. Very little information is publicly available to gauge industry attitudes, and industry players refused to participate in the study. Media-reporting creates the impression that SSB taxation would raise several concerns, such as employment losses [40]. Some respondents indicated that industry’s response to tackling NCDs was lacklustre as there is no perceived economic benefit to them getting involved.

Academics are well placed to assist in evidence-based initiatives to tackle NCDs. However, academic respondents reported that they had limited abilities (or the perception of limited abilities) to influence policy-makers.

## Discussion

The majority of the Namibian population is marginalized, being of low socioeconomic status. For many, economic emancipation is a priority over healthy eating. In most cases, they cannot afford the high costs of healthy foods, nor do they have access to a variety of choices [41]. These contextual challenges have negatively affected the implementation of policies and the attainment of expected outcomes towards the prevention and control of NCDs in Namibia. Related to this, despite high-level policy stating the need for a gendered lens on NCDs, no specific policy response has been formulated. In addition to the policy gaps, there is a dearth of evidence on the gender burden of NCDs.

Namibia demonstrates political will to address the problem of NR-NCDs, at a high level. However, despite establishing a multisectoral approach, policy documents directly addressing NCDs are primarily the responsibility of the health sector. Specific interventions for NR-NCDs in sectors outside of health appear to be less likely to be implemented; for example, an SSB tax has not been introduced. In contrast to SSB taxation, the Namibian Government successfully enacted legislation and regulations to control the consumption of other substances that are risk factors for NCDs, such as alcohol (Liquor Act 6 of 1998) and smoking (Tobacco Products Control Act, Act No. 1 of May 2010).

Our study also found that translation of policy priorities such as SSB taxation into action was hampered by the lack of Government resources, which is likely to be compounded by a shrinking economy and various competing interests [42]. In other countries and cities [15,43,44], SSB taxation has been framed as an opportunity to increase revenue (funding) for implementation of Government (health) policies that currently lack funding. This was not raised by respondents or found in existing policy documents, and is a gap in the existing advocacy narrative in Namibia for SSB taxation. For example, SSB taxation revenue in Namibia could be used to support production of healthy local traditional food alternatives, to complement the disincentive that SSB taxation provides with positive incentives (increased availability, reduced price) for healthy foods.

Geo-political influences are expected to impact on Namibia’s attitude towards an SSB tax. When South Africa adopts a novel mechanism or policy, local policymakers are heavily influenced by the perceptions of the policy’s success in South Africa. Sometimes, the adoption of outside policy is at the expense of understanding and analysing local factors, as witnessed in Brazil and other Latin American countries [45]. Given the fluidity of movement of people and products between countries, including South African non-alcoholic beverage imports into Namibia, and the influence of the Southern African Customs Union, which attempts to homogenise elements of industry, including taxation, in southern Africa, a regional perspective to advocacy approaches to SSB taxation would be of value.

### Policy implications

Although there is policy endorsement of an SSB tax in Namibia, this has not been translated into enactment of the tax. In our analysis of the Kingdon stream, the ‘problems’ of SSB txation and NCDs are not well captured in the existing data sources. For this reason, policy formulation, action and evaluation could be enhanced by improved empirical evidence. The lack of detailed data on NR-NCDs and their prevalence could hinder evidence-based responses to NCDs and make it difficult to translate international recommendations into policy action. The use of international guidelines and direction from international treaties without country-specific context, results in potentially inappropriate targets, which are difficult to monitor and evaluate for their impact and success [46]. There is therefore a need for national studies to better understand NR-NCDs in Namibia, support the adoption of an SSB tax, and evaluate the potential impact of an SSB tax.

### Recommendations

There is a lack of data related to SSB taxation and this deficit extends to evaluating perceptions and potential industry responses to issues such as the SSB tax, which makes assessing the politics stream difficult. It is difficult to gauge whether a reluctance to introduce the tax is based on industry narratives, purely industry influence, or apprehensions that emanate from other sources. This information is necessary to, among others, formulate advocacy responses. In addition, there is a need for strong pro-tax advocacy and messaging to support the adoption of the tax and generate public support for the intervention, as was done in South Africa [47]. Further, in Namibia, while the problem of NR-NCDs is well recognised, SSBs as a specific contribution to the problem of NR-NCDs is less well understood. A benchmarking study should be initiated to understand the barriers to, and best practice for, the implementation of SSB taxation.

### Limitations

This study contributes to the limited literature on the NCD prevention policy landscape and the political economy of SSB taxation in Namibia. We utilised a mixture of documentary data analysis, key informant interviews, and paid data sets to analyse the policy landscape as comprehensively as possible. However, a key limitation is that there is limited information on the SSB and SSB-related industries. Publicly available information on industries and industry activity was limited, and industry representatives declined to participate in this study. Even with purchased data sets, our ability to analyse actors whose economic interests may be harmed by an SSB tax was limited.

## Conclusion

The Government of Namibia has taken positive steps towards addressing NCDs, but several obstacles remain. The gap between high-level policy and its diffusion into sectoral and more specific interventions was apparent. This was found for SSB taxation, which has policy endorsement but is not in the stage of enactment. Industry attitudes and approaches are largely undocumented, which hinders effective advocacy for an SSB tax. Namibia’s trade relations and proximity to South Africa make a regional case for SSB taxation advocacy compelling.

## Paper context

The prevalence of nutrition-related non-communicable diseases in Namibia is high. An evidence-based countermeasure is sugar-sweetened beverage taxation. In this study, we examined the barriers to, and possible facilitators of, introducing such a tax in Namibia. We identified a high degree of political will, but a lack of information on several fronts as a barrier to policy formulation and adoption. Our findings indicate that more resources should be invested in producing evidence to support policy action.
